# The High Osmolarity Glycerol (HOG) Pathway Functions in Osmosensing, Trap Morphogenesis and Conidiation of the Nematode-Trapping Fungus *Arthrobotrys oligospora*

**DOI:** 10.3390/jof6040191

**Published:** 2020-09-27

**Authors:** Chih-Yen Kuo, Sheng-An Chen, Yen-Ping Hsueh

**Affiliations:** 1 Molecular and Cell Biology, Taiwan International Graduate Program, Academia Sinica and Graduate Institute of Life Sciences, National Defense Medical Center, Taipei 11490, Taiwan; yenkuo@gate.sinica.edu.tw; 2 Institute of Molecular Biology, Academia Sinica, Nangang, Taipei 11490, Taiwan; dexter_samuel199@hotmail.com

**Keywords:** *Arthrobotrys oligospora*, high osmolarity glycerol (HOG) pathway, Hog1 mitogen-activated protein kinase, osmolarity, nematocidal activity, signaling mucin Msb2

## Abstract

Hog1, a mitogen-activated protein kinase (MAPK), has been identified in diverse fungal species, and it regulates various cellular processes, such as osmoadaptation, nutrient-sensing, and pathogenesis. However, the roles that Hog1 plays in nematode-trapping fungi were previously unclear. Here, we characterized orthologs of *Saccharomyces cerevisiae* Hog1 and membrane mucin Msb2 in the nematode-trapping fungus *Arthrobotrys oligospora*. We generated gene deletion mutants of *HOG1* and *MSB2* in *A. oligospora*, and characterized their roles in osmosensing, growth, and trap morphogenesis. We found that both *hog1* and *msb2* mutants were highly sensitive to high osmolarity. Predation analyses further revealed that *hog1* and *msb2* deletion caused a reduction in trap formation and predation efficiency. Furthermore, *HOG1* is required for conidiation in *A. oligospora*, demonstrating its critical role in this developmental pathway. In summary, this study demonstrated that the conserved Hog1 and Msb2 govern physiology, growth and development in the nematode-trapping fungus *A. oligospora*.

## 1. Introduction

The ability to sense and respond appropriately to the environmental changes is essential for any organism. For example, eukaryotic cells regulate their internal osmolarity in response to osmolarity changes in the environments. In the budding yeast *Saccharomyces cerevisiae*, cells respond to increases in external osmolarity by increasing intracellular glycerol synthesis [1]. In the fungal kingdom, osmosensing has been linked to fungal development, metabolism and virulence [2]. In some plant pathogens, such as *Fusarium graminearum* and *Botrytis cinerea*, the pathway regulating responses to hyperosmotic stress is also involved in hyphal development and pathogenesis [3,4].

Carnivorism is universal in the animal kingdom; however, certain plants and fungi that inhabit in low-nutrient environments have also evolved the ability to prey on small animals such as insects and nematodes. To access nitrogen resources, the Venus flytrap, *Dionaea muscipula*, attracts prey by emitting of volatile organic compounds that simulate food odors [5]. Similarly, a number of fungi, known as nematode-trapping fungi (NTF), can develop complex trapping structures to capture and kill nematodes [6,7]. Trap formation in NTF is mainly induced by different biotic and abiotic factors as well as direct contact between fungal hyphae and living nematodes [8].

Mitogen-activated protein kinase (MAPK) signaling cascades function as key and evolutionarily conserved signal transducers in all eukaryotes [9]. Sequential activation of MAPK cascades ultimately results in transcription factor up-regulation and expression of specific sets of genes in response to environmental stimuli [10]. One of the best-studied MAPK pathways is the high osmolarity glycerol (HOG) pathway in the model yeast *S. cerevisiae*, which responds to changes in external osmolarity. The *S. cerevisiae* MAPK, Hog1, is a central player in this pathway, controlling various osmoadaptive responses. Operating upstream of Hog1 are two sensor proteins, Sln1 and Sho1, that act independently but in a functionally redundant manner. Sln1 is a transmembrane histidine kinase, whereas Sho1 is a tetraspanning membrane protein [11,12]. Furthermore, the Sho1 signaling cascade itself also involves two functionally redundant osmosensors, Hkr1 and Msb2 (Supplementary Material Figure S1) [13,14,15,16]. Concomitant activation of Hog1 thus controls an array of osmoadaptive responses. Although the Hog1 MAPK pathway is mainly involved in osmoregulation in *S. cerevisiae*, its orthologs in various other fungi often exert additional biological functions [2,10]. For instance, in *Neurospora crassa*, this MAPK cascade is involved in nutrient sensing [17], and it is essential for virulence in several plant and human fungal pathogens [3,18,19,20,21]. 

In this study, we investigated the roles of Hog1 MAPK pathway in *Arthrobotrys oligospora*, one of the most common and best understood species of NTF [22]. We demonstrate that both Hog1 and Msb2 are involved in the pathogenicity, and that Hog1 is required for conidiation. Our *msb2* mutant line shared several but not all phenotypes displayed by our *hog1* mutant. Consequently, we speculate that additional upstream osmosensors, which are functionally redundant to Msb2, regulate Hog1 activation. In summary, the Hog1 MAPK pathway appears to be important for the predator–prey interactions between NTF and nematodes. 

## 2. Materials and Methods 

### 2.1. Identification and Deletion of the KU70, HOG1 and MSB2 Homologs in A. oligospora

The *KU70* homologs of *A. oligospora* were identified in Blast2GO 5 Pro [23] by performing a local BLAST analysis. We used the amino acid sequences of Ku70 orthologs from *S. cerevisiae* (UniProt ID P32807) as the queries and the proteome of the *A. oligospora* TWF154 strain as the database. The same method was used to identify the *HOG1* and *MSB2* homologs of *A. oligospora*. The amino acid sequences of Hog1 and Msb2 orthologs from *Aspergillus nidulans* (UniProt ID Q9P419, Q5AXD9), *Neurospora crassa* (Q96TL5), *F. graminearum* (P0C431, I1RNP9), and *S. cerevisiae* (P32485, P32334) were used as the queries.

*KU70*, *HOG1* and *MSB2* were deleted by means of a homologous recombination-based strategy, which reported previously for *A. oligospora* [24,25]. Primers used for generating the *KU70*, *HOG1* and *MSB2* deletion mutants are described in the Supplementary Materials.

### 2.2. Generation of Target Gene Deletion Mutants

In a previous study [26], we generated deletion mutants via homologous recombination and determined that the homologous recombination rate in *A. oligospora* is extremely low (~3%). Therefore, here, we generated a non-homologous end-joining-deficient strain (*ku70*) to increase the efficiency of targeted genes deletion in fungi [27,28,29]. An overlap PCR-based construct (donor DNA) was obtained by fusing the 1.5 kb long sequences flanking the open reading frames of the target genes (amplified from genomic DNA of the *A. oligospora* TWF154 strain) to the hygromycin-B resistance cassette (amplified from vector pAN7-1 [30]). The construct was introduced into protoplasts of the *A. oligospora* TWF154 strain via PEG-mediated transformation. Generation of protoplasts in *A. oligospora* was carried out based on previously described protocols [26]. For transformations, ~10^6^ protoplasts were gently mixed with 5 µg of donor DNA and incubated on ice for 30 min, after which five volumes of PTC (40% *w/v* PEG 4000, 10 mM Tris-HCl pH 7.5, 50 mM CaCl_2_) were added and incubated at room temperature for 20 min. Then, 50 mL of molten regeneration agar (0.3% *w/v* yeast extract, 0.3% *w/v* casein, 20.5% *w/v* sucrose) at 45 °C containing 100 µg/mL hygromycin B was added to the protoplast mixture and poured into Petri dishes. After 7 days at 25 °C, transformants grown on selective medium were screened for gene replacement by rapid genomic extraction and PCR. Briefly, a small cluster of aerial hyphae was added to PCR tubes containing 30 µL of DNA TE buffer (10 mM Tris-HCl pH 8, 0.5M EDTA pH 8), which were placed in a microwave and heated for 2 min. After the samples cooled down to room temperature, they were re-heated in the microwave. Finally, the samples were placed in a −20 °C refrigerator to cool down for 10 min. Two µL of the samples were then used as template for PCR reactions. 

For deletion of the *A. oligospora HOG1* gene (*EYR41_010451*) and *MSB2* (*EYR41_007180*), we fused the overlap PCR-based construct with flanking the open reading frame of the target gene to a nourseothricin sulfate resistance cassette (amplified from vector pRS41N [31]). The construct was introduced into protoplasts of the *ku70* (control) strain via PEG-mediated transformation. For transformations, ~10^5^ protoplasts were used and the concentration of nourseothricin sulfate was 250 µg/mL.

### 2.3. Phenotypical Characterization

To quantify trap formation, fungal isolates were grown on low nutrient medium (LNM) for 5 days and then transferred to a fresh LNM plate for a further 48 h (25 °C, dark). Thirty N2 *Caenorhabditis elegans* L4-stage larvae were then introduced onto the fungal plates for 6 h, after which the animals were removed by washing with sterile water. Twenty-four hours after exposure, micrographs were taken of fungi using a Nikon SMZ 745T stereo microscope. Three images were randomly taken of each fungal plate using 40× magnification, and the sum of all traps in the three images was recorded. 

To estimate survival rates of *C. elegans* upon exposure to *hog1* and *msb2* mutant lines, 80 young adult stage N2 *C. elegans* were introduced onto mutant fungal plates for 6 h. Numbers of moving nematodes were assessed every 2 h for a total of 12 h using a stereo microscope.

### 2.4. Statistics

For trap quantification analysis, a two-tailed unpaired Student’s t test was performed to determine the statistical difference between the control and experimental samples using GraphPad Prism 8. For survival rate analysis, a two-tailed unpaired Student’s t test was conducted to determine the statistical difference between the control and experimental groups after being exposed to *C. elegans* for 12 h. A value of *p* < 0.05 was considered statistically significant.

## 3. Results

### 3.1. Identification of the KU70 Gene in A. oligospora

The homologous recombination rate in *A. oligospora* was extremely low (~3%) in a previous study [26]. To enhance the recombination rate, we generated a non-homologous end-joining-deficient strain (*ku70*) which has been reported to increase the efficiency of gene deletion in fungi [27,28,29]. We surveyed the genome of *A. oligospora* strain TWF154 [26] to search for the orthologs of the *S. cerevisiae KU70* gene, and identified *EYR41_000878* (*KU70*).

**Figure 1:**
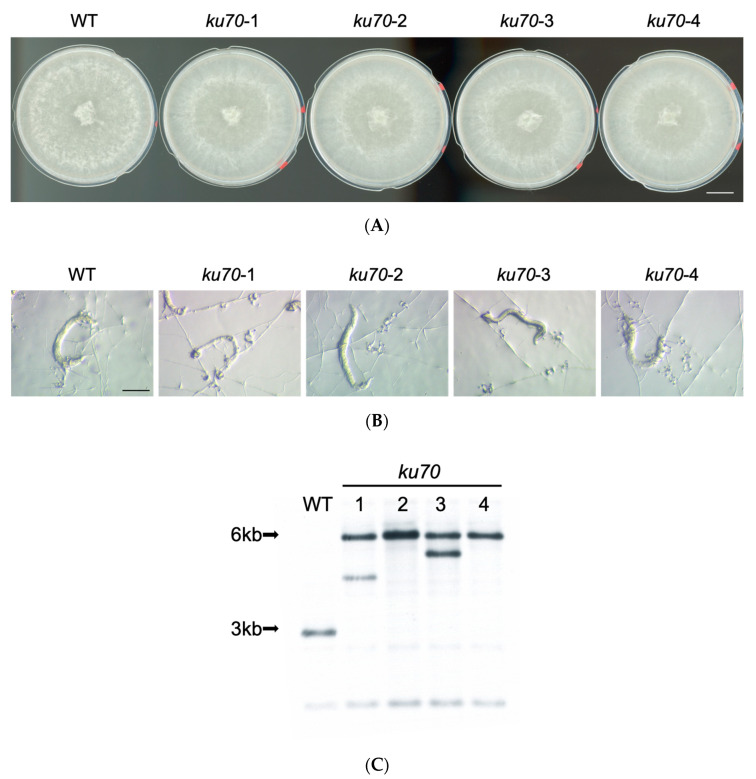
Phenotypic characteristics in a non-homologous end-joining-deficient strain (*ku70*) of *A. oligospora*. (**A**) Colonies of TWF154 (WT) and independent *ku70* strains grown on potato dextrose agar (PDA) plates (5-cm diameter) for 4 day at 25 °C (Scale bar, 1 cm). (**B**) Representative brightfield images of the traps induced by N2 *C. elegans* in the TWF154 (WT) and independent *ku70* mutant strains. Images were taken 24 h after induction with N2 *C. elegans* (Scale bar, 200 μm). (**C**) Southern blot confirmation of *KU70* deletion. The wild-type TWF154 strain displayed a predicted band of size 2.9 kb, whereas *ku70* mutants had predicted bands of 5.9 kb.

The *ku70* mutants did not exhibit any obvious growth defects under normal growth conditions (Figure 1A). To examine if the deletion of *KU70* in *A. oligospora* influenced trap morphogenesis, we compared phenotypic differences between the TWF154 (WT) and the *ku70* lines upon exposure to *C. elegans*. We observed that independent *ku70* mutants formed traps and exhibited nematocidal activity comparable to that of the wild-type strain (Figure 1B). Southern blot analysis revealed that some *ku70* deletion strains we obtained contained multiple ectopic integrations of the repair DNA (Figure 1C). Therefore, we decided to conduct gene disruption experiments in the *ku70-2* mutant line to facilitate homologous recombination. 

### 3.2. Identification of the HOG1 and MSB2 Genes in A. oligospora Suggests a Conserved Role in Osmosensing

**Figure 2:**
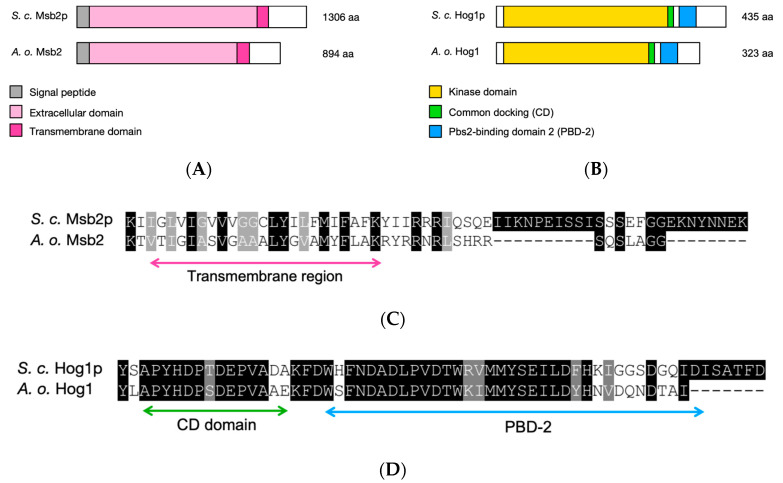
*A. oligospora* Msb2 and Hog1 are structural orthologs of the *S. cerevisiae* Msb2p and Hog1p. (**A**) Schematic representation of the domain structure of *A. oligospora* and *S. cerevisiae* Msb2 proteins. Both proteins share common features of signaling mucins, including a N-terminal signal peptide, a large extracellular domain, and one transmembrane that is closed to the C-terminus. (**B**) Schematic representation of the domain structure of *A. oligospora* and *S. cerevisiae* Hog1 proteins. Both proteins share common features, including a protein kinase domain, common docking (CD) domain and Pbs2-domain binding 2 (PBD-2). (**C**) Amino acid sequence alignment of the transmembrane region of the *S. cerevisiae* Msb2 (S. c. Msb2p) and *A. oligospora* Msb2. (**D**) Amino acid sequence alignment of the CD domain and PBD-2 of the *S. cerevisiae* Hog1 (S. c. Hog1p) and *A. oligospora* Hog1. Highly conserved residues are shaded in black; moderately conserved residues are shaded in gray.

We were interested in examining the role of the HOG MAPK pathway in responses of *A. oligospora* to *C. elegans* presence. First, we surveyed the genome of *A. oligospora* strain TWF154 [26] to search for the orthologs of the *S. cerevisiae HOG1* and *MSB2* genes, and identified two targets, i.e., *EYR41_010451* (*HOG1*) and *EYR41_007180* (*MSB2*), respectively. The overall sequence identities between *S. cerevisiae* and *A. oligospora* Msb2 and Hog1 proteins were ~44.5% and ~54.8%, respectively. The predicted *A. oligospora* Msb2 protein has a domain architecture similar to that of *S. cerevisiae* Msb2 (Figure 2A,C), including an N-terminal signal sequence (22 amino acids), a large extracellular domain (amino acids 23 to 793), and one transmembrane domain (TM; 23 amino acids). *A. oligospora* Hog1 also has a similar domain structure to that of *S. cerevisiae* Hog1 (Figure 2B,D), including a highly conserved protein kinase domain (amino acids 1 to 272), a common docking (CD) domain (amino acids 273 to 284) and a Pbs2-domain binding 2 (PBD-2) domain (amino acids 290 to 320). Both the CD domain and the PBD-2 are required for the activation of Hog1 by Pbs2 in *S. cerevisiae* [32]. These results indicate that these two genes might play important roles, including pathogenesis, in nematode-trapping fungi. We therefore generated targeted gene deletion mutants for *HOG1* and *MSB2* mutants via a homologous recombination-based strategy reported previously for *A. oligospora* [24,25]. The deletion of *HOG1* or *MSB2* genes was confirmed by Southern blot analysis (Supplementary Material Figure S1).

**Figure 3:**
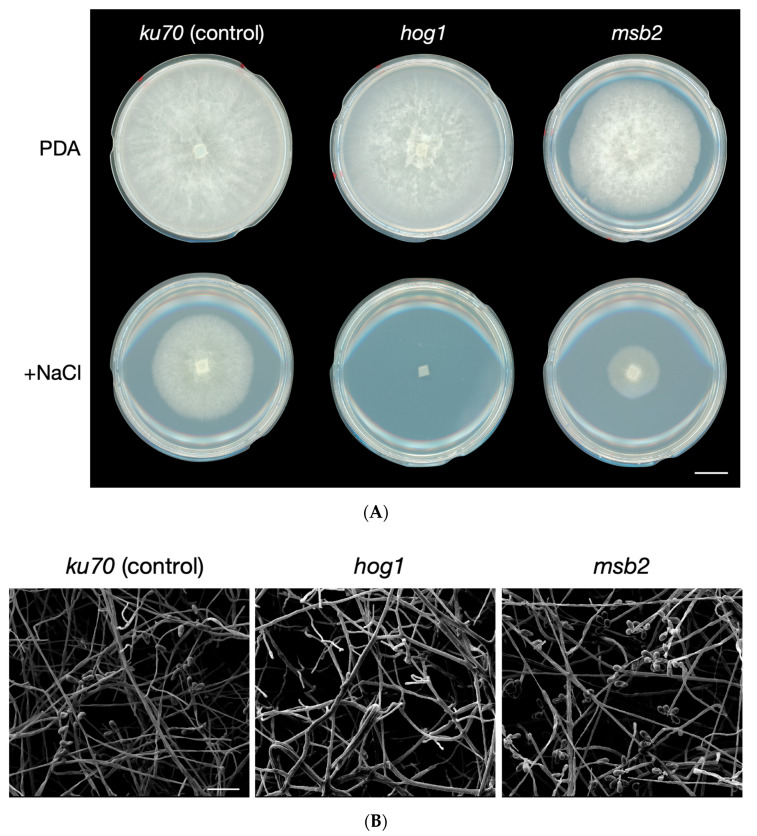
Defects of the *hog1* and *msb2* mutants in response to hyperosmotic stress. (**A**) Colonies of *ku70* (control), *hog1*, and *msb2* mutants grown on PDA plates (5-cm diameter) for 4 days with or without 1% (*w*/*v*) NaCl (Scale bar, 1 cm). (**B**) SEM images of *ku70* (control), *hog1*, and *msb2* mutants after growing on PDA plates (5-cm diameter) for 4 days (Scale bar, 50 μm).

The HOG MAPK pathway is important in regulating responses to hyperosmotic stress in fungi [2], so we investigated the effect of treating our *A. oligospora hog1* and *masb2* mutants with excess sodium chloride (NaCl). On potato dextrose agar (PDA) with 1% (*w/v*) NaCl, the *hog1* mutant had not grown after incubation for 4 days, whereas the *msb2* mutant showed a growth defect relative to the *ku70* control (Figure 3A). These results indicate that the osmoregulatory role of the HOG MAPK cascade is conserved in *A. oligospora.* Given that the *msb2* mutant exhibited only a slight growth defect in the presence of excess NaCl, it is plausible that Msb2 is not the only receptor operating upstream of the HOG pathway.

The *hog1* mutant did not exhibit any obvious growth defects under normal growth conditions (Figure 3A), but a more detailed morphological analysis using scanning electron microscopy (SEM) revealed a defect in conidial formation (Figure 3B). In contrast, though the *msb2* mutant presented reduced growth in PDA alone, its conidiation was as robust as the wild-type (Figure 3B). Thus, Hog1 appears to play a role in osmoregulation and the asexual development of *A. oligospora*. In contrast, Msb2 appears to control hyphal growth and to participate in osmoregulation.

### 3.3. The Hog1 Pathway Is Required for Proper Formation of Traps during Nematode Induction

**Figure 4:**
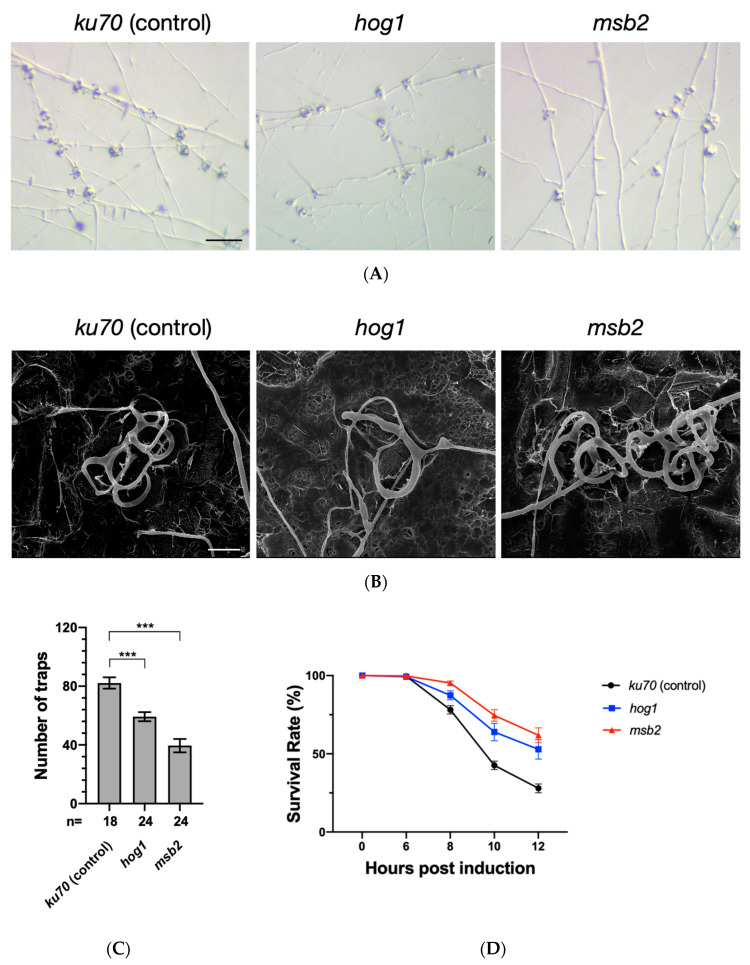
The HOG1 MAPK pathway is involved in proper formation of traps during nematode induction. (**A**) Representative brightfield images of the traps induced by N2 *C. elegans* in the *ku70* (control), *hog1*, and *msb2* mutants. (Scale bar, 200 μm). (**B**) Representative SEM images showing defective trap network formation by the *hog1* mutant. (Scale bar, 25 μm). (**C**) Quantification of the trap numbers induced by N2 *C. elegans* for the *ku70* (control), *hog1*, or *msb2* mutant lines. (Mean ± SEM; n shown along the x axis; asterisks represent significance levels of unpaired t test compared to the control. *** *p* < 0.001.) (**D**) Survival rates of nematodes upon exposure to the *ku70* (control), *hog1*, or *msb2* mutant lines. (Mean ± SEM; *n* = 14).

To examine if the HOG pathway functions in *A. oligospora* trap morphogenesis, we compared phenotypic differences between the control and the mutant fungal lines upon exposure to *C. elegans*. We observed that both *hog1* and *msb2* mutants formed traps, but trap numbers were reduced when compared to the control (Figure 4A). Quantification of trap morphogenesis revealed that the trap numbers in the *hog1* and the *msb2* mutant reduced to ~70% and ~50% of the *ku70* control, respectively (Figure 4C). Next, we conducted SEM analysis to further examine the detail trap morphology in the wild-type and mutant strains. We observed that the *hog1* mutant exhibited defective development of three-dimensional trap structures; however, the traps formed by the *msb2* mutant were comparable to those of the *ku70* control (Figure 4B).

Both *hog1* and *msb2* mutants developed fewer traps upon *C. elegans* exposure; therefore, we evaluated their trapping efficiency. In the *ku70* control, only ~28% of the nematodes applied to *A. oligospora* fungal plates had survived after 12 h (Figure 4D). In contrast, survival rates of nematodes exposed to the *hog1* and *msb2* mutants were ~50 to 60% (Figure 4D). Together, the reduction in trap numbers and the development of inadequate trapping structures demonstrate that the *hog1* and *msb2* mutants display impaired predation ability when compared to the wild-type.

## 4. Discussion

NTF hold great potential to be utilized as biological control agents in agricultural settings. However, the interactions between nematodes and NTF have not been investigated in detail at the molecular level. In recent years, a number of studies have begun to uncover the molecular mechanisms underlying the biology of these specialized predators. For example, it has been reported that two MAP kinases, Slt2 in the cell wall integrity (CWI) pathway and Ime2 in the inducer of meiosis 2 (Ime2) pathway, are involved in *A. oligospora* trap formation and nematocidal activity [33,34]. These data suggest that MAPK pathways play important roles in interaction between NTF and their prey. 

**Figure 5:**
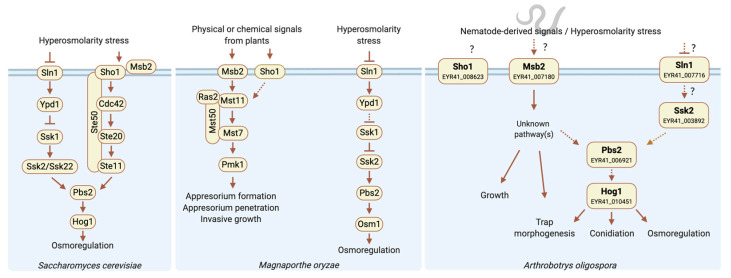
Schematic models for the role of Hog1 and Msb2 in yeast and filamentous fungi. *S. cerevisiae* Sln1, Sho1, and Msb2 sense the changes in the osmolarity of the extracellular environment and activate Hog1 for osmoregulation. The proteins separated by a slash symbol (/) are functionally redundant. Not all of the known components are shown. In blast fungus, *M. oryzae*, Msb2 and Sho1 are involved in recognizing physical or chemical signals from plant to activate the downstream Mst11-Mst7-Pmk1 MAPK cascade which is responsible for pathogenesis. The Ssk2-Pbs2-Osm1 MAPK cascade is activated by Sln1 to respond to changes in the osmolarity of the extracellular environment. In *A. oligospora*, both Hog1 and Msb2 are not only involved in osmoadaptation but also in growth, trap morphogenesis and conidiation. Solid and dashed arrows indicate the verified and putative connections, respectively. The figure was created with BioRender.com.

The Hog1 MAPK pathway is thought to be primarily involved in osmoregulation in *S. cerevisiae*, whereas its orthologs in various filamentous fungi often have additional biological functions [2,10]. In Figure 5, we illustrated schematic models for the role of Hog1 and Msb2 playing in *S. cerevisiae*, *M. oryza*, and *A. oligospora*. In *S. cerevisiae*, functionally redundant osmosensors activate downstream Hog1 MAPK to respond to changes in osmolarity in the extracellular environment [13,15]. In *M. oryzae*, Osm1 (ortholog of Hog1) activated by upstream osmosensor Sln1 plays a critical role in osmoregulation but is dispensable for plant infection [35]. In contrast, *M. oryzae* Msb2 activates Pmk1, an ortholog of the *S. cerevisiae* Fus3 MAPK-mediating mating process, to regulate appressorium formation and penetration [36]. In other plant pathogens, such as *Ustilago maydis*, deletion of *MSB2* also impedes the differentiation of appressoria on inductive surfaces [37]. In *Fusarium oxysporum*, which does not produce appressoria, mutants lacking Msb2 present reduced penetration of host roots [38]. These findings indicate that Msb2 has evolved diverse functions in different fungal species.

In *A. oligospora*, we found that the *hog1* and the *msb2* mutants were hypersensitive to osmotic stress, especially for the *hog1* mutant that was completely inhibited when excess NaCl was present in the culture medium. In some pathogenetic fungi, deletion of *HOG1* has been shown to reduce conidia numbers [20,39], and we also found that our *A. oligospora hog1* mutant was defective in conidial formation. Thus, Hog1 not only participates in the osmoregulation, but also regulates asexual reproduction in *A. oligospora*.

Both the *hog1* and the *msb2* mutants were capable of forming traps upon exposure to *C. elegans,* but in both cases, trap numbers were reduced. Notably, the *hog1* mutant displayed a defect in developing proper three-dimensional trap structures, although those of the *msb2* mutant were comparable to *ku70* control traps. These observations potentially explain the decreased nematocidal activity of our mutants, as assessed by *C. elegans* survival. 

Our *msb2* mutant line shared several but not all of the phenotypes displayed by the *hog1* mutant. Accordingly, we speculate that additional functionally redundant osmosensors also regulate Hog1 activation (Figure 5), a scenario that has also been described for the HOG pathway of budding yeast [13,14]. Besides, in *U. maydis* and *M. oryzae*, it has been reported that compared to individual deletion mutants of *SHO1* or *MSB2*, the *sho1 msb2* double mutant completely abolished appressorium formation [36,37]. Therefore, we hypothesize that *SHO1* and *SLN1*, which are both present in the genome of *A. oligospora*, likely also function as upstream osmosensors. Overall, our study demonstrates that the Hog1 MAPK pathway displays a conserved role in osmoregulation, and is also critical in regulation of conidiation and trap morphogenesis in the NTF *A. oligospora*.

## Supplementary Materials

The following are available online at https://www.mdpi.com/2309-608X/6/4/191/s1, Figure S1: Southern blot analysis of targeted gene deletion mutants generated in the *ku70* background, Table S1: Primer used in this study.
