# Supplementary material for: The High Osmolarity Glycerol (HOG) Pathway Functions in Osmosensing, Trap Morphogenesis and Conidiation of the Nematode-Trapping Fungus Arthrobotrys oligospora
Source: J Fungi (Basel). 2020 Sep 27;6(4):191. doi: 10.3390/jof6040191 (PMC7711997; doi:10.3390/jof6040191)
Supplement: Supplementary file 1 [file jof-06-00191-s001.zip › jof-941364-supplementary.docx]

Supplemental Materials and Methods:

**Figure S1**

**A B**

**
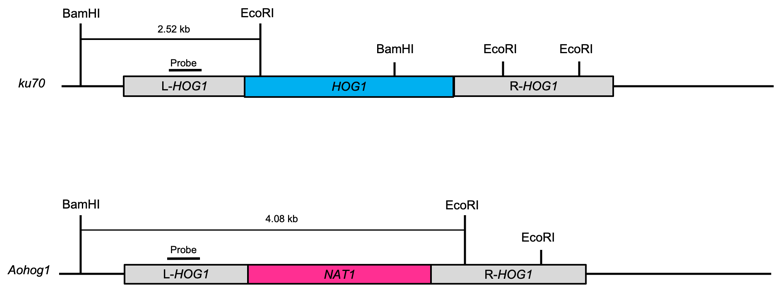

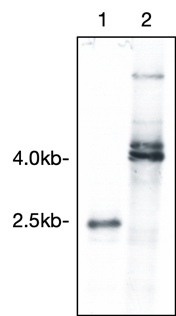
**

**C D**

**
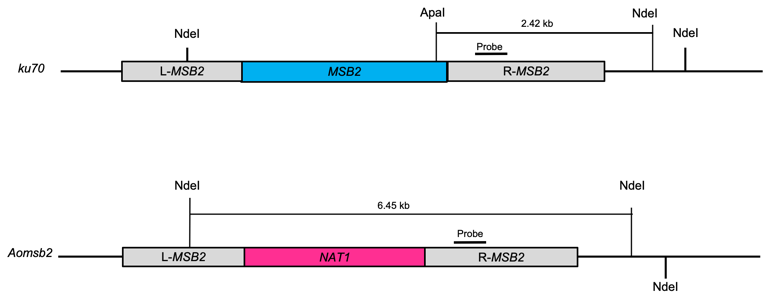

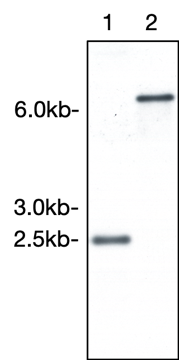
**

**Figure S1.** Southern blot analysis of targeted gene deletion mutants generated in the *ku70* background. (A) Restriction map of the *HOG1* region and the position of the fragment used as a probe. (B) Total DNA from the *A. oligospora* *ku70* (control; lane 1) and *hog1* mutant (lane 2) lines was digested using BamHI and EcoRI restriction enzymes and hybridized with the probe. The *ku70* strain generated a 2.52-kb band that matches the expected control banding pattern. The *hog1* mutant displayed a 4.08-kb band matching the expected *hog1*-deletion banding pattern. (C) Restriction map of the *MSB2* region and the position of the fragment used as a probe. (D) Total DNA from the *ku70* (control; lane 1) and *msb2* mutant (lane 2) lines was digested using ApaI and NdeI restriction enzymes and hybridized with the probe. The *ku70* strain displayed a 2.42-kb band that matches the expected control banding pattern. The *msb2* mutant had a 6.45-kb band matching the expected *msb2*-deletion banding pattern.

Supplementary Table 1. Primer used in this study.

| Primers | Sequence (5’🠢3’) | Description |
| --- | --- | --- |
| 298 | CGAGCTCCCAAATCTGTCCA | Amplification of HygB resistance cassette from vector pAN7-1; (forward) |
| 299 | GTGTACCTGTGCATTCTGGG | Amplification of HygB resistance from vector pAN7-1; (reverse) |
| 614 | CGGCATCAGAGCAGATTGTAC | Amplification of clonNAT resistance cassette from vector pRS41N; (forward) |
| 615 | GGTATTTCACACCGCACAGG | Amplification of clonNAT resistance cassette from vector pRS41N; (reverse) |
| 1067 | GTATGGGCTAAATGTACGGG | NAT1 insertion check (reverse) |
| 1068 | GGTCAGGTTGCTTTCTCAGG | NAT1 insertion check (forward) |
| 1069 | TGTCCAAGCAGCAAAGAGTG | HygR insertion check; (forward) |
| 1070 | TTTCATACACCGGGCAAAGC | HygR insertion check; (reverse) |
| 2188 | TCTGGAAGGTGGGAAAGTCG | *KU70* deletion in *A.o* (5' flanking, forward) |
| 2189 | TGGACAGATTTGGGAGCTCGCAAGTTGAGGCTGGTGGAAG | *KU70* deletion in *A.o* (5' flanking, reverse) |
| 2190 | CCCAGAATGCACAGGTACACGGTTGAGCGGATTGAAGAACAC | *KU70* deletion in *A.o* (3' flanking, forward) |
| 2191 | AGAAGGCTATTGCCGGGAAC | *KU70* deletion in *A.o* (3' flanking, reverse) |
| 2192 | GGATATACCGGATGCACCGAG | *KU70* deletion check (internal, forward) |
| 2193 | GCGATGAACCATGCGATACC | *KU70* deletion check (internal, reverse) |
| 2194 | AGCCTCGGTCTGCTTTCTTG | *KU70* deletion check (cassette integration, forward) |
| 1433 | CCTGGGCGATGTTGAGAAATTG | *MSB2* knockout check (internal, forward) |
| 1434 | TCGAAGTAGTTTGCGGAAGGTC | *MSB2* knockout check (internal, reverse) |
| 1435 | GCATACTACGGAAGACGGTTCG | *MSB2* knockout check (cassette integration, forward) |
| 2136 | TGTCAGTAAGTACAGCACACAGC | *MSB2* knockout in *A.o* (5' flanking, forward) |
| 2137 | GTACAATCTGCTCTGATGCCGCCTTACGAGCGACCTTGGAG | *MSB2* knockout in *A.o* (5' flanking, reverse) |
| 2138 | CCTGTGCGGTGTGAAATACCCCAGCGTTTGTTCAGCCTTAA | *MSB2* knockout in *A.o* (3' flanking, forward) |
| 2139 | AACCTGGGCATTCGGTCTAG | *MSB2* knockout in *A.o* (3' flanking, reverse) |
| 2286 | GGAGGACGGTGATTGACGTTG | *HOG1* knockout in *A.o* (5' flanking, forward) |
| 2287 | GTACAATCTGCTCTGATGCCGAGGAGGAGGAGGAGTAGGAAAG | *HOG1* knockout in *A.o* (5' flanking, reverse) |
| 2288 | CCTGTGCGGTGTGAAATACCGACAAGGCGAAAGGGCAAAC | *HOG1* knockout in A.o (3' flanking, forward) |
| 2289 | TGCAGGTTGATGGAATCGAGAG | *HOG1* knockout in A.o (3' flanking, reverse) |
| 2290 | TCACAAGCAGGTTCGTAGTCC | *HOG1* knockout check (internal, forward) |
| 2291 | TAGCGACAAATATCCAATCGGAGG | *HOG1* knockout check (internal, reverse) |
| 2292 | TTGGTGCTAGATGATGGTGACG | *HOG1* knockout check (cassette integration, forward) |

| 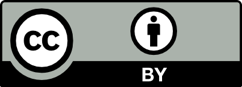 | © 2020 by the authors. Submitted for possible open access publication under the terms and conditions of the Creative Commons Attribution (CC BY) license (http://creativecommons.org/licenses/by/4.0/). |
| --- | --- |
